# A meta-analysis of the effects of multimodal intervention measures on the recovery of postpartum women after cesarean section

**DOI:** 10.3389/fmed.2025.1690139

**Published:** 2025-12-17

**Authors:** Zhi-Min Zhao, Jia-Qi Yang, Gui-Xian Wang, Xiao-Jing Yu, Yun Feng

**Affiliations:** 1 Department of Anesthesia and Surgery, Zhejiang Hospital, Hangzhou, China; 2 Department of Obstetrics, Zhejiang Hospital, Hangzhou, China

**Keywords:** cesarean section, multimodal intervention, enhanced recovery, postoperative pain, early mobilization, meta-analysis

## Abstract

**Object:**

This systematic review and meta-analysis evaluated whether multimodal intervention bundles improve recovery after cesarean section compared with routine care.

**Methods:**

Following PRISMA 2020, we searched PubMed, Embase, Scopus, Web of Science, and major Chinese databases (CNKI, Wanfang, VIP) from inception to July 2025 without language restrictions. Eligible randomized controlled trials enrolled postpartum women undergoing cesarean section and implemented bundles comprising ≥2 distinct domains (e.g., opioid-sparing analgesia, early mobilization, early oral intake/nutrition or breastfeeding support, structured nursing, psychological education). The primary endpoint was pain intensity at 24 h (VAS); secondary endpoints included pain at other time points, analgesic consumption, time to first ambulation and first flatus, adverse events, length of stay, breastfeeding initiation/time, and maternal psychological recovery. Relative risks were pooled for dichotomous outcomes and mean (or standardized mean) differences for continuous outcomes, using random effects when heterogeneity was substantial.

**Results:**

Twelve RCTs (*n* = 1,497), all conducted in China, met inclusion. Multimodal care reduced 24 h pain versus routine care (MD − 0.96 on a 0–10 VAS; 95% CI − 1.28 to −0.64; I^2^ = 97%), shortened time to first ambulation (MD − 4.58 h; 95% CI − 6.32 to −2.84; I^2^ = 95%) and to first flatus (MD − 2.28 h; 95% CI − 2.57 to −1.98; I^2^ = 97%), and lowered overall adverse events (RR 0.28; 95% CI 0.19–0.41; I^2^ = 0%). No significant difference was observed for time to first breastfeeding (MD 1.39 h; 95% CI − 3.86 to 6.64; I^2^ = 99%; *p* = 0.60). Risk of bias was generally moderate to high owing to limited blinding; Egger’s test for the primary outcome did not suggest publication bias.

**Conclusion:**

Collectively, the current evidence suggests that multimodal, ERAS-oriented care pathways may facilitate safer and more rapid early recovery following cesarean section, particularly by enhancing analgesic efficacy, mobilization, and gastrointestinal function. Nevertheless, the marked heterogeneity in bundle composition, implementation intensity, and outcome definitions, together with the single-country evidence base and moderate risk of bias, substantially limits the certainty and generalizability of these findings.

## 1 Introduction

Cesarean section is one of the most frequently performed surgical procedures worldwide and its absolute numbers continue to rise (1, 2). Although cesarean delivery is often lifesaving for the mother or fetus, the surgery and the immediate postoperative period pose distinctive challenges for recovery: women must rapidly regain physical function while simultaneously initiating breastfeeding, bonding, and newborn care (3). Conventional, single-modality postoperative routines, centered on intermittent opioid analgesia, delayed mobilization, and conservative feeding, have important limitations, including inadequate pain control during activity, opioid-related adverse effects, delayed gastrointestinal recovery, and inconsistent support for early ambulation and lactation (4, 5). These factors can prolong convalescence, increase complications, and undermine patient experience in the early postpartum period.

Enhanced Recovery After Surgery (ERAS) programs address such limitations through coordinated, evidence-informed bundles that target multiple biological and behavioral pathways in parallel (6, 7). When adapted to obstetric care, multimodal interventions typically integrate opioid-sparing analgesia (e.g., neuraxial techniques and non-opioid adjuncts), criteria-based early mobilization, early oral intake with prudent fluid therapy, structured nursing protocols, psychological education and expectation setting, and breastfeeding support (8–10). Optimized pain control enables earlier mobilization, which in turn improves pulmonary hygiene and accelerates bowel recovery; early oral intake with prudent fluid therapy further promotes gastrointestinal motility; proactive nursing surveillance and structured education lower preventable adverse events; and tailored counseling reduces anxiety and strengthens maternal self-efficacy for newborn care.

Despite growing adoption of multimodal, ERAS-like pathways after cesarean section, the reported effects across trials remain variable (11). Heterogeneity in intervention composition (number, intensity, and timing of components), perioperative practices, and outcome definitions have yielded discrepant estimates for core endpoints such as pain intensity, time to ambulation, gastrointestinal recovery, complications, length of stay, initiation of breastfeeding, and maternal psychological status. Prior reviews have often focused on single components (e.g., analgesic techniques) rather than the cumulative effect of bundled care, or they have synthesized limited subsets of outcomes, leaving uncertainty about the overall magnitude and consistency of benefit attributable to multimodal strategies in the postpartum cesarean population.

To elucidate the impact of the composite construct of “multimodal intervention” on early postoperative recovery, safety, and patient-centered outcomes, a comprehensive, focused synthesis of evidence is warranted. By evaluating intervention bundles that explicitly integrate at least two distinct domains—analgesia, early mobilization, nutrition/feeding, psychological education, and nursing—we can estimate the overall clinical effect of coordinated care compared with usual practice or single-component comparators and identify outcome domains in which uncertainty persists. Accordingly, we conducted a systematic review and meta-analysis of randomized controlled trials (and closely related designs) to quantify the effects of multimodal interventions on recovery after cesarean delivery, with the aim of providing an integrated evidence base to guide the design, implementation, and iterative refinement of multimodal pathways in obstetric practice.

## 2 Methods

### 2.1 Protocol and process

This meta-analysis was conducted in accordance with the PRISMA 2020 guidelines for systematic reviews and meta-analyses. The research question was formulated according to the PICO framework: the population included postpartum women undergoing cesarean section; the intervention consisted of multimodal measures comprising at least two distinct domains (e.g., psychological support, optimized analgesia, early mobilization, nutritional support, breastfeeding support, enhanced recovery nursing); the comparator was routine care or single-component interventions; the outcomes included a primary endpoint of postoperative pain intensity (VAS score) at 24 h (10, 12), while secondary endpoints included VAS scores at 12 and 48 h, postoperative analgesic consumption, time to first ambulation and bowel function recovery, incidence of postoperative complications, length of hospital stay, breastfeeding initiation or rate, and maternal psychological recovery.

Pre-specified rules for data selection and extraction were defined. When multiple postoperative time points for VAS were reported, the time point closest to 24 h (±6 h) was chosen as the primary analysis indicator. If both resting and activity VAS were available at the same time point, the activity VAS was prioritized. For multi-arm trials, comparisons were made between the multimodal intervention group and the routine care group; if multiple eligible intervention groups existed, they were combined to avoid double counting.

### 2.2 Eligibility criteria

Studies were eligible for inclusion if they met the following criteria: (i) study design was a randomized controlled trial (RCT); high-quality quasi-experimental studies were considered in sensitivity analyses if essential outcome data were available; (ii) participants were postpartum women undergoing cesarean section (elective or emergency), aged 18 years or older; (iii) the intervention group received multimodal interventions, which were defined as any combination of at least two distinct domains from the listed categories, for example, analgesia and psychological support, early mobilization and nutritional care, or breastfeeding support and nursing interventions; (iv) the comparator was usual care or a single-component intervention; and (v) at least one relevant outcome was reported, including postoperative pain intensity (VAS), analgesic consumption, time to first ambulation or bowel recovery, length of hospital stay, incidence of complications, breastfeeding outcomes, or maternal psychological status.

Exclusion criteria were: (i) non-clinical studies such as reviews, case reports, conference abstracts, or expert opinions; (ii) interventions not clearly described or not meeting the definition of multimodal interventions, for example studies involving a single analgesic method only; (iii) participants not limited to women undergoing cesarean section; (iv) studies without a control group or with insufficient outcome data for analysis; and (v) duplicate publications or overlapping datasets, in which case only the most recent or most complete study was included.

### 2.3 Information sources and search strategy

We systematically searched the following databases: PubMed, Embase, Scopus, Web of Science, CNKI, Wanfang, and VIP. The search covered all records from database inception to July 2025, with no language restrictions, to ensure a comprehensive inclusion of relevant studies. In addition to database searches, we performed manual searches of reference lists of included studies, relevant reviews, and gray literature sources (e.g., theses, clinical trial registries) to identify additional eligible studies. The search strategy combined controlled vocabulary terms (MeSH/Emtree) and free-text terms using Boolean operators. Core search terms included “cesarean section,” “postpartum women,” “multimodal intervention,” “enhanced recovery,” “pain management” and “rehabilitation.” The full list of search terms and Boolean combinations used for each database is provided in the Supplementary material.

### 2.4 Study selection

All records identified through the database search were imported into EndNote for duplicate removal. Two reviewers independently screened all records in two stages: an initial screening of titles and abstracts to exclude clearly irrelevant studies, followed by a full-text review to assess eligibility based on the predefined inclusion and exclusion criteria. Any discrepancies between the two reviewers were resolved through discussion, and if disagreement persisted, a third reviewer adjudicated. The number of studies identified, excluded, and finally included will be presented in a PRISMA flow diagram, which details the overall search yield, number of duplicates removed, reasons for exclusion, and the final number of studies included.

### 2.5 Data extraction and management

Two reviewers independently extracted data using a standardized data extraction form to ensure accuracy and consistency. Extracted information included: first author, year of publication, country/region, study design, sample size, baseline characteristics of participants (e.g., age, Gestational weeks), details of multimodal interventions and their components, comparator interventions, follow-up duration, and all pre-specified outcomes, such as VAS scores, time to first ambulation and bowel function recovery, incidence of complications and incidence of adverse events (nausea, vomiting, dizziness, urinary retention, excessive uterine bleeding, fever, and wound complications). For continuous variables, means and standard deviations were extracted whenever available. When only medians and interquartile ranges were reported, established formulas were used to approximate means and standard deviations. For dichotomous variables, the number of events and total participants were extracted. If data were incomplete or unclear, attempts were made to contact the corresponding authors for clarification; if unsuccessful, data were estimated using alternative methods such as digitizing published figures. All extracted data were cross-checked by a third reviewer, and any discrepancies were resolved by discussion or consensus within the research team to ensure data integrity and reliability.

### 2.6 Risk of bias assessment

The methodological quality of randomized controlled trials was assessed using the risk of bias tool recommended in the Cochrane Handbook for Systematic Reviews of Interventions. Seven domains were evaluated: (i) random sequence generation; (ii) allocation concealment; (iii) blinding of participants and personnel; (iv) blinding of outcome assessment; (v) completeness of outcome data; (vi) selective reporting; and (vii) other potential sources of bias. Each domain was judged as having “low risk,” “high risk,” or “unclear risk” of bias based on the reported information. Two reviewers independently performed the risk of bias assessment, with disagreements resolved by discussion or adjudication by a third reviewer when necessary. The results will be summarized in both tabular and graphical formats, showing the risk of bias for each study across domains and the overall distribution of bias risk. For any included non-randomized studies, the ROBINS-I tool was used to assess the risk of bias.

### 2.7 Statistical analysis

All statistical analyses were performed using Review Manager (RevMan, Version 5.4). For dichotomous outcomes (e.g., incidence of complications), results were expressed as relative risk (RR) with 95% confidence intervals (CI). For continuous outcomes (e.g., VAS scores, time to ambulation), standardized mean difference (SMD) with 95% CIs was used. Heterogeneity among studies was assessed using Cochrane’s Q test and the I^2^ statistic. Significant heterogeneity was considered present when *p* < 0.10 or I^2^ > 50%. A random-effects model was applied in the presence of significant heterogeneity, while a fixed-effects model was used otherwise. Publication bias was assessed using Egger’s regression test. All statistical tests were two-sided, and a *p*-value <0.05 was considered statistically significant.

## 3 Results

### 3.1 Study selection

**Figure 1:**
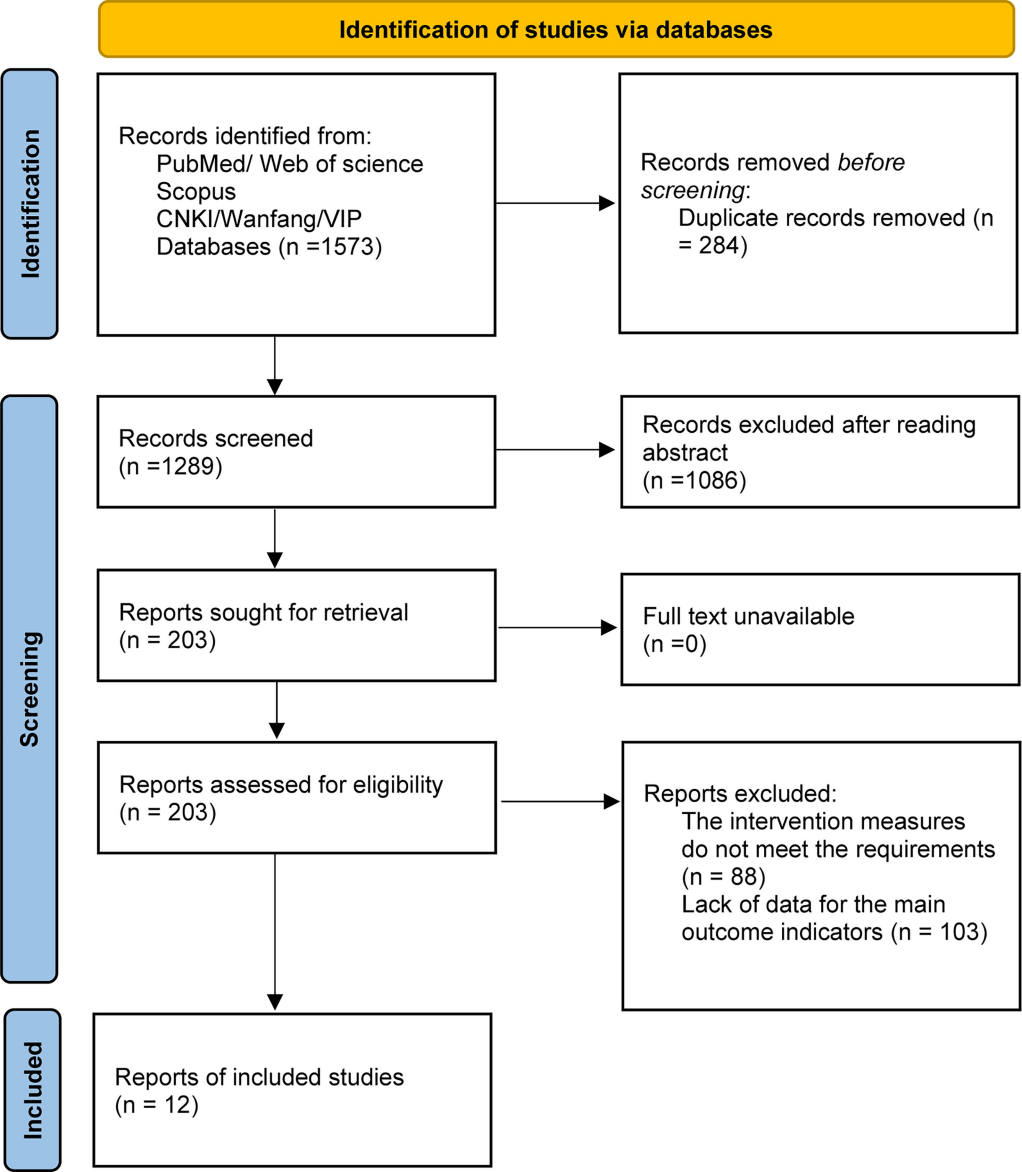
PRISMA flow diagram of the literature search and study selection process.

A total of 1,573 records were initially identified through database searches. After removing 284 duplicates, 1,289 records remained for title and abstract screening, of which 1,086 were excluded as irrelevant. The full texts of 203 articles were assessed for eligibility, with no unavailability. After full-text assessment, 191 studies were excluded, including 88 that did not meet the definition of multimodal interventions and 103 that lacked data on the main outcome indicators (Figure 1). Ultimately, 12 studies met the inclusion criteria and were included in the meta-analysis.

### 3.2 Study characteristics

A total of 12 studies (8–10, 12–20) were included, all conducted in China and published between 2018 and 2025. Sample sizes ranged from 60 to 600 participants, with a total of 1,497 women enrolled. All included studies were RCTs, conducted exclusively in China (Table 1), all studies integrated multimodal preventive analgesia—such as intraoperative–postoperative bridging with patient-controlled epidural analgesia (PCEA) and VAS-guided graded pain control—with early ambulation, early oral intake, psychological support, or structured nursing care, reflecting an ERAS-oriented multimodal approach.

**Table 1:** Basic characteristics of the included studies.

Study	Year	Research type	Region/Country	Methods of intervention		Primary outcome	Secondary outcome
				I	C		
Kuang, C (8)	2023	RCT	China	Multimodal analgesic care: team-based management, preoperative PCEA education and assessment, intraoperative ropivacaine + sufentanil analgesia, postoperative VAS ≤ 3, early mobilization and psychological support.	Routine care: health education, psychological support, lifestyle and breastfeeding guidance, basic analgesia, and pain monitoring.	VAS at 24 h	Time to first ambulation, Time to first breastfeeding and Incidence of adverse events
Lu, H (9)	2018	RCT	China	Multimodal analgesic care: team-based management, preoperative PCEA education and assessment, intraoperative ropivacaine + sufentanil analgesia, postoperative VAS ≤ 3, early mobilization and psychological support.	Routine postoperative analgesia care, including standard PCEA guidance, pain assessment (VAS ≤ 3), basic health education, and psychological support.	VAS at 24 h	Time to first ambulation, Time to first breastfeeding and Incidence of adverse events
Lu, L (10)	2022	RCT	China	Multimodal analgesic care: team-based management (APS including anesthesiologists, obstetricians, and nurses), preoperative PCEA education and assessment, intraoperative ropivacaine + sufentanil analgesia, individualized adjustment within 24–48 h to maintain postoperative VAS ≤ 3, early ambulation, uterine contraction care, and psychological support.	Routine care: standard PCEA use and guidance, pain assessment (VAS ≤ 3), health education on diet, rest, and breastfeeding, and psychological support.	VAS at 24 h	N/A
Sun, D (12)	2019	RCT	China	Multimodal analgesic care: team-based management (anesthesiologists, obstetricians, and nurses), preoperative PCEA education and assessment, intraoperative ropivacaine + sufentanil analgesia, individualized adjustment within 24–48 h to maintain postoperative VAS ≤ 3, early ambulation, uterine contraction management, and continuous psychological support.	Routine care: standard PCEA use and guidance, pain assessment (VAS ≤ 3), general postpartum nursing (diet, activity, breastfeeding), and basic psychological support.	N/A	Time to first ambulation, Time to first flatus, Time to first breastfeeding and Incidence of adverse events
Wang, H (13)	2023	RCT	China	Multimodal analgesic care: team-based management (anesthesiologists, obstetricians, and nurses), preoperative PCEA education and assessment, intraoperative ropivacaine + sufentanil analgesia, individualized adjustment within 24–48 h to maintain postoperative VAS ≤ 3, early ambulation, uterine contraction care, and psychological support.	Routine care: standard PCEA use and guidance, pain assessment (VAS ≤ 3), health education on diet, rest, and breastfeeding, and psychological support.	VAS at 24 h	Time to first ambulation, Time to first flatus and Time to first breastfeeding
Wang, K (14)	2021	RCT	China	Multimodal analgesic care: team-based management (anesthesiologists, obstetricians, and nurses), preoperative education on PCEA and postoperative pain prevention, intraoperative ropivacaine + sufentanil analgesia, individualized adjustment within 24–48 h to maintain VAS ≤ 3, early mobilization, uterine contraction management, and continuous psychological and emotional support.	Routine care: standard postoperative nursing, health education on diet, rest, and breastfeeding, routine PCEA use and guidance, pain assessment (VAS ≤ 3), and psychological support.	VAS at 24 h	Time to first ambulation, Time to first flatus
Yang, J (15)	2024	RCT	China	Multimodal analgesic care: team-based management (anesthesiologists, obstetricians, and nurses), preoperative education on PCEA and pain coping strategies, intraoperative ropivacaine + sufentanil analgesia, individualized adjustment within 24–48 h to maintain VAS ≤ 3, early ambulation, emotional counseling, and uterine contraction management.	Routine care: conventional analgesic management, health education on diet, rest, and breastfeeding, routine PCEA guidance, pain assessment (VAS ≤ 3), and psychological support.	N/A	Time to first ambulation, Time to first flatus and Time to first breastfeeding
Yang, Y (16)	2023	RCT	China	Multimodal warming care: team-based management (anesthesiologists, obstetricians, and nurses); preoperative temperature assessment and patient education; intraoperative multimodal warming combining warm-air blanket, warmed infusion (37–38 °C), and radiant heat; continuous temperature monitoring during and after surgery; early mobilization, emotional support, and neonatal thermal protection measures.	Routine care: standard intraoperative temperature management (maintaining room temperature at 26–28 °C, humidity 60–70%), conventional fluid warming, and postoperative observation of body temperature recovery.	N/A	Time to first flatus and Incidence of adverse events
Ye, X (17)	2022	RCT	China	Multimodal analgesic care: team-based management (anesthesiologists, obstetricians, and nurses), preoperative analgesia education and psychological counseling, intraoperative ropivacaine + sufentanil PCEA analgesia, individualized adjustment to maintain VAS ≤ 3 within 24–48 h, early mobilization, uterine contraction care, emotional support, and continuous monitoring for pain relief effectiveness and adverse reactions.	Routine care: standard postoperative analgesic management, conventional PCEA use and monitoring, pain assessment (VAS ≤ 3), and basic health education on rest, diet, and breastfeeding.	VAS at 24 h	Time to first ambulation and Time to first breastfeeding
Zheng, Q (18)	2021	RCT	China	Multimodal warming care: team-based management; combined use of active warming blanket, warmed infusion fluids (37.0–38.0 °C), and pre-warmed surgical drapes; early intraoperative warming initiated immediately after entering the OR; continuous temperature monitoring at multiple body sites (core, fetal, and peripheral); postoperative temperature recording and comfort assessment.	Routine warming care: operating room temperature maintained at 22–25 °C and humidity at 45–60%; infusion and irrigation fluids warmed to ~37 °C; passive warming only with standard measures.	N/A	Time to first ambulation and Incidence of adverse events
Zhu, J (19)	2024	RCT	China	Multimodal rehabilitation care: team-based management (obstetricians, anesthesiologists, and nurses); preoperative education and anxiety relief; intraoperative ropivacaine + sufentanil analgesia; individualized PCEA adjustment within 24–48 h; early mobilization (initial ambulation within 6–8 h post-surgery, gradually increasing to 30 m walking within 24 h); continuous emotional support, breastfeeding guidance, and evaluation of recovery indicators (VAS, SAI, PSQI).	Routine care: conventional postoperative management, standard PCEA use and monitoring, pain assessment (VAS ≤ 3), health education, and psychological comfort measures.	N/A	Incidence of adverse events
Zhu, M (20)	2025	RCT	China	Multimodal FTS-based care: team-based management integrating obstetricians, anesthesiologists, and nurses; preoperative psychological counseling and blood pressure stabilization; intraoperative warming and analgesia optimization; early postpartum mobilization and guided breastfeeding; close hemodynamic monitoring and fluid management; comprehensive education for self-care and postpartum blood pressure control.	Routine care: standard obstetric management, conventional intraoperative and postoperative monitoring, pain assessment (VAS ≤ 3), and basic health education.	VAS at 24 h	N/A

### 3.3 Risk of bias assessment

**Figure 2:**
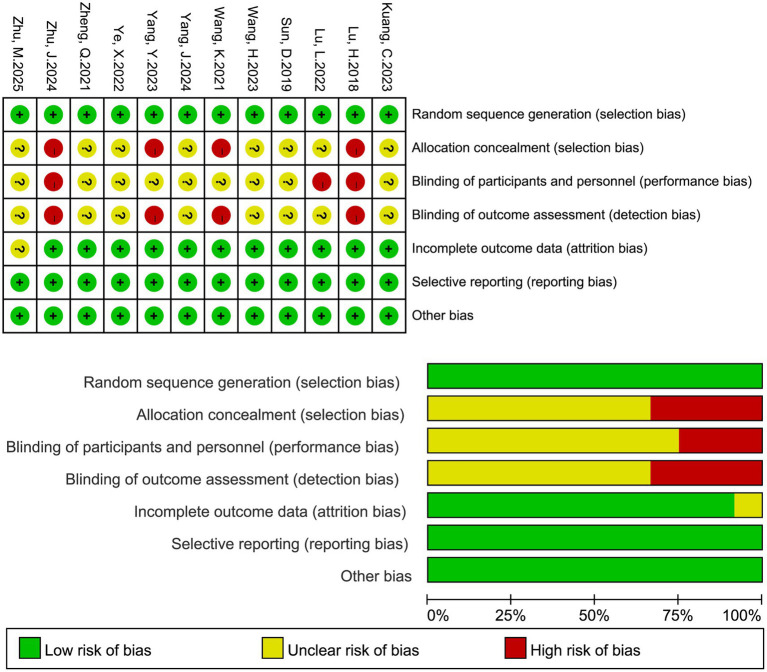
Risk of bias assessment of the included studies.

All 12 included studies were randomized controlled trials (Figure 2). According to the Cochrane risk of bias tool, most studies were judged to be at low risk in terms of random sequence generation and completeness of outcome data. Several studies did not provide sufficient details regarding allocation concealment and were therefore rated as unclear risk. With respect to blinding of participants and personnel and blinding of outcome assessments, many trials did not implement or did not clearly report blinding procedures, leading to judgments of high or unclear risk. A few studies showed potential concerns about selective reporting, but the overall impact was limited. In summary, the methodological quality of the included studies was considered moderate to high, with the main limitation being the lack of adequate blinding.

### 3.4 Main results

#### 3.4.1 Postoperative pain score (VAS) at 24 h

**Figure 3:**
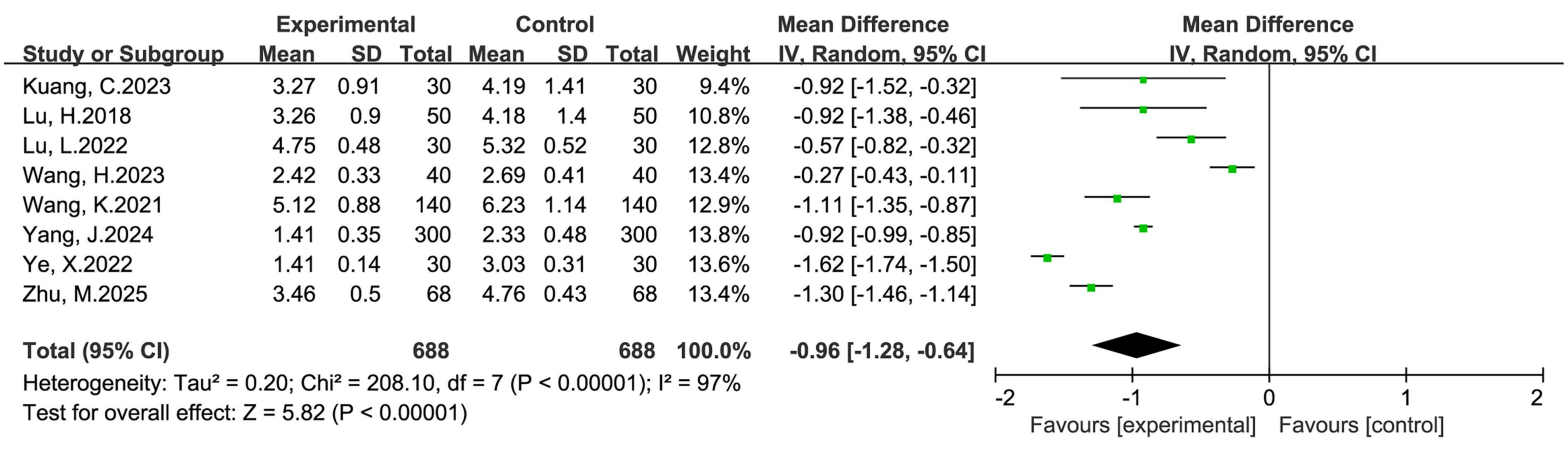
Forest plot of VAS at 24 h after cesarean section: multimodal vs. routine care.

Eight studies involving a total of 1,376 women (688 in the intervention group and 688 in the control group) reported VAS scores at 24 h. Among the included studies, there was variation in how VAS was reported: some specified rest or activity, while others did not clarify the context. Minor differences in time points (within the 24 ± 6 h range) also existed. These were accounted for in the analysis, and activity VAS was prioritized when both were available. The pooled analysis demonstrated that multimodal interventions significantly reduced pain intensity compared with routine care (MD = −0.96, 95% CI: −1.28 to −0.64, *p* < 0.00001). Heterogeneity across studies was high (I^2^ = 97%), and thus a random-effects model was applied (Figure 3). Despite substantial heterogeneity, the direction of effect consistently favored multimodal interventions, supporting their beneficial role in postoperative pain management.

#### 3.4.2 Time to first ambulation

**Figure 4:**
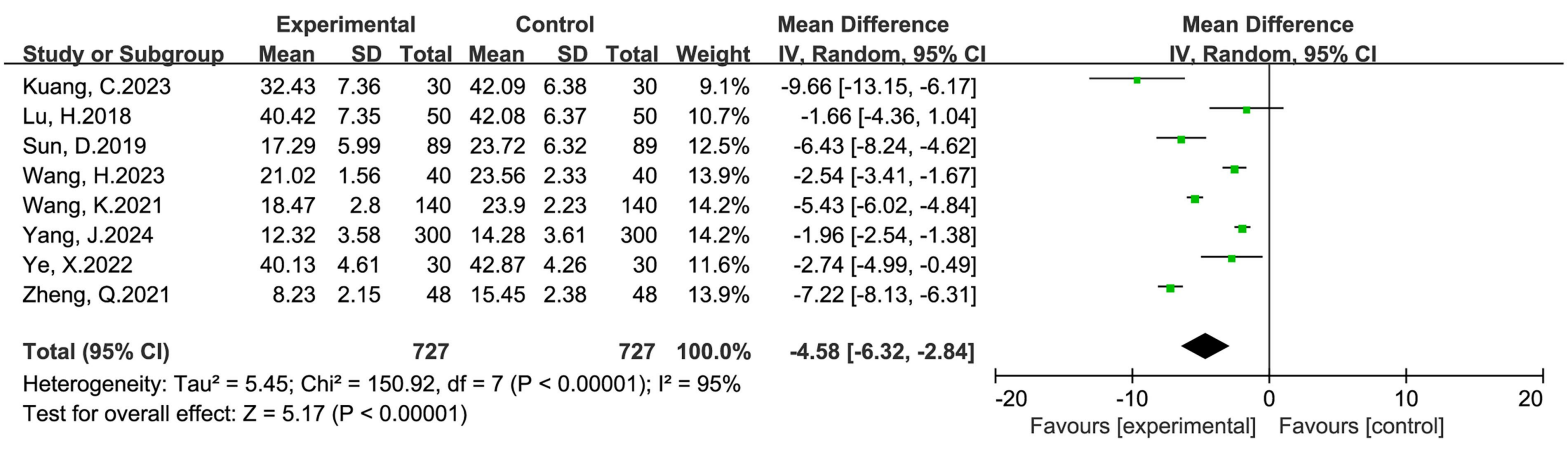
Forest plot of time to first ambulation (hours) after cesarean section: multimodal vs. routine care.

Eight studies involving 1,454 women (727 in the intervention group and 727 in the control group) reported time to first ambulation. Pooled results showed that multimodal interventions significantly reduced the time to first ambulation compared with routine care (MD = −4.58 h, 95% CI: −6.32 to −2.84, *p* < 0.00001). Heterogeneity was high (I^2^ = 95%), so a random-effects model was applied (Figure 4). Despite variability, all studies consistently demonstrated earlier mobilization in the multimodal intervention group.

#### 3.4.3 Time to first flatus

**Figure 5:**
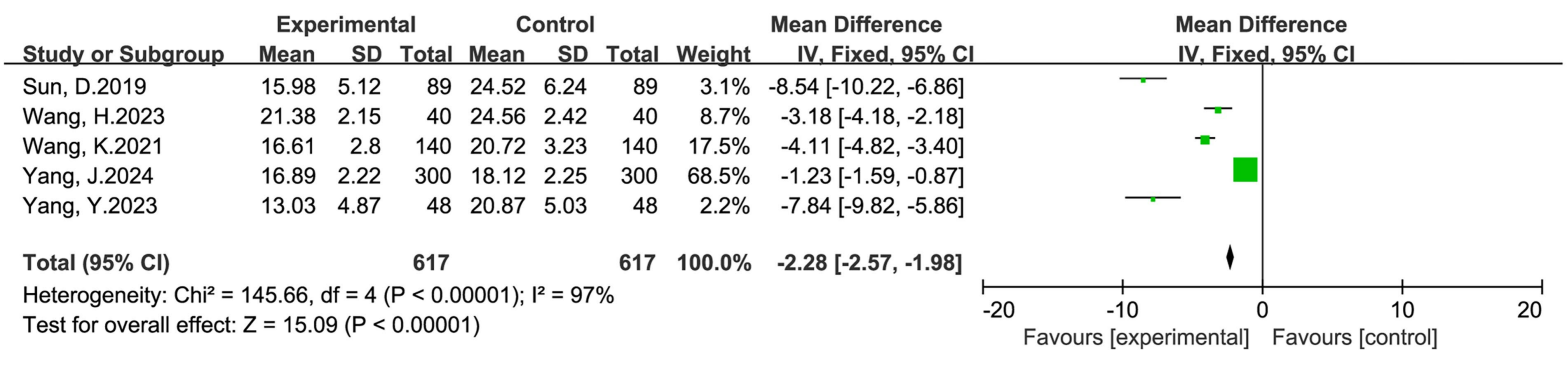
Forest plot of time to first flatus (hours) after cesarean section: multimodal vs. routine care.

Five studies involving 1,234 women (617 in the intervention group and 617 in the control group) reported time to first flatus. The pooled results indicated that multimodal interventions significantly shortened the time to first flatus compared with routine care (MD = −2.28 h, 95% CI: −2.57 to −1.98, *p* < 0.00001). Heterogeneity was high (I^2^ = 97%), so a random-effects model was applied (Figure 5). Despite variability, the overall results consistently supported earlier gastrointestinal recovery in the multimodal intervention group.

#### 3.4.4 Time to first breastfeeding

**Figure 6:**
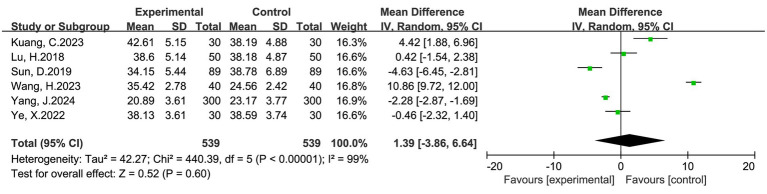
Forest plot of time to first breastfeeding (hours) after cesarean section: multimodal vs. routine care.

Six studies including 1,078 women (539 in the intervention group and 539 in the control group) reported time to first breastfeeding. The pooled analysis showed no statistically significant difference between multimodal interventions and routine care (MD = 1.39 h, 95% CI: −3.86 to 6.64, *p* = 0.60). Heterogeneity was extremely high (I^2^ = 99%), and thus a random-effects model was applied (Figure 6). The direction of individual study results varied, with some favoring earlier breastfeeding under multimodal interventions and others showing no clear difference. Overall, current evidence is insufficient to confirm the consistent benefit of multimodal interventions on earlier initiation of breastfeeding.

#### 3.4.5 Incidence of adverse events

**Figure 7:**
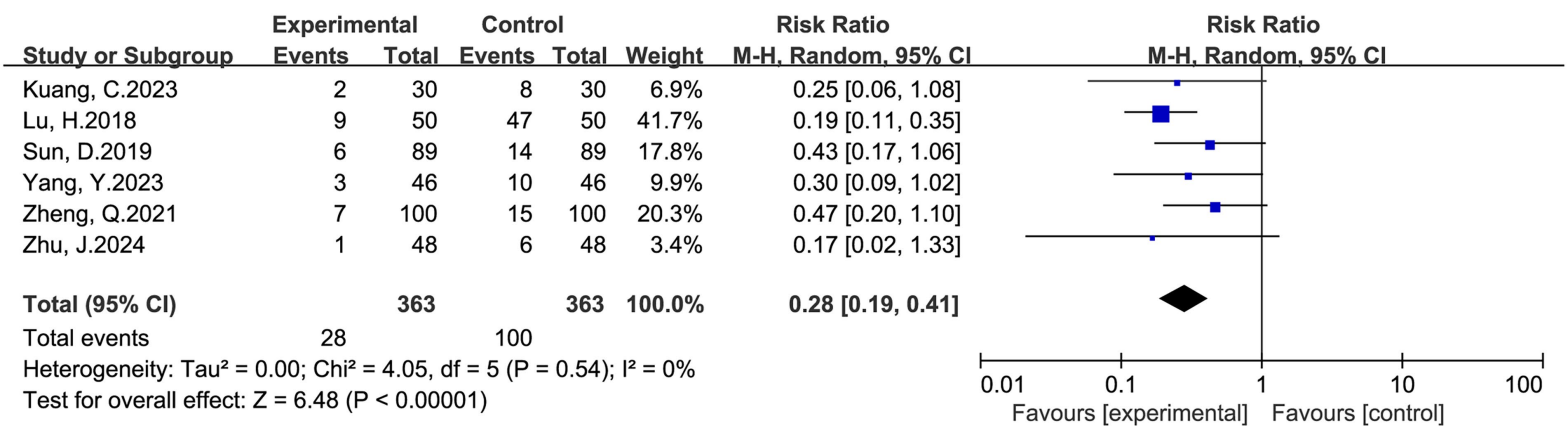
Forest plot of adverse event incidence after cesarean section: multimodal vs. routine care.

Six studies including 726 women (363 in the intervention group and 363 in the control group) reported the incidence of postoperative adverse events. The pooled analysis demonstrated that multimodal interventions significantly reduced the overall incidence of adverse events (RR = 0.28, 95% CI: 0.19–0.41, *p* < 0.00001). No significant heterogeneity was observed (I^2^ = 0%), suggesting robust and consistent findings across studies (Figure 7). The overall trend consistently favored multimodal interventions in reducing postoperative complications.

#### 3.4.6 Psychological recovery

Psychological recovery was predefined as one of the secondary outcomes. However, none of the included studies provided sufficient or comparable quantitative data (e.g., standardized psychological assessment scores) to enable a pooled analysis. As a result, this outcome could not be statistically analyzed.

### 3.5 Publication bias

For the primary outcome (VAS at 24 h), Egger’s regression test was performed. The Egger’s test indicated no significant publication bias (*p* > 0.05).

## 4 Discussion

In this meta-analysis of 12 randomized trials encompassing 1,497 women undergoing cesarean section, multimodal intervention bundles that integrated elements such as optimized opioid-sparing analgesia, early mobilization, nutritional or feeding support, structured education/psychological counseling, and enhanced recovery nursing produced clinically meaningful gains across several core recovery domains. Compared with routine care, multimodal pathways reduced pain intensity at 24 h by nearly one VAS unit (MD − 0.96), advanced the time to first ambulation by roughly 4–6 h (MD − 4.58 h), and shortened time to first flatus by approximately 2–3 h (MD − 2.28 h). Importantly, these programs were associated with a substantial and homogeneous reduction in postoperative adverse events (RR 0.28; I^2^ = 0%), indicating that accelerated recovery was achieved without compromising safety. By contrast, pooled effects on the time to first breastfeeding were inconsistent and statistically non-significant, suggesting that standard multimodal bundles may not systematically influence this outcome.

Multimodal, opioid-sparing analgesia can reduce nausea, sedation, and ileus risk, enabling earlier mobilization, improved pulmonary hygiene, and greater maternal engagement in newborn care (9, 12). Earlier ambulation, in turn, supports thromboembolism prevention and gastrointestinal motility, while proactive nursing surveillance and structured education likely reduce complications through earlier detection and mitigation of issues such as urinary retention, dizziness, and wound problems (21). The coherence of improvements across pain, mobility, and gut function—coupled with a robust safety signal—argues for genuine synergistic effects of bundled care rather than isolated gains from any single component (18, 19).

By contrast, the neutral effect on time to first breastfeeding likely reflects heterogeneity in outcome measurement and clinical context rather than a true lack of efficacy of multimodal care. Across studies, the timing anchor and definition of effectiveness were not uniform (e.g., measuring from birth, completion of uterine closure, arrival in the PACU, or ward admission; criteria ranging from “attempted latch” to “sustained, effective suckling”) (15, 17, 19), which increases variance and dilutes any true effect. Institutional policies and workflows—such as the implementation of immediate skin-to-skin contact (SSC), continuity of rooming-in, and the availability of lactation consultants (particularly overnight and on weekends) (22)—together with handoff and transfer timing between the operating and recovery areas, often determine the feasibility of early breastfeeding. In addition, analgesic strategies and maternal readiness (23) (e.g., neuraxial opioid–related sedation/nausea, residual motor block, orthostatic intolerance) and neonatal factors (late preterm status, transient respiratory adaptation, NICU observation) can objectively narrow the window for early latching (24). Accordingly, this null finding should not be taken to indicate that multimodal pathways are ineffective; rather, it likely suggests that most bundles provided limited coverage of steps directly related to breastfeeding. In practice environments with greater emphasis on SSC, continuous rooming-in, and accessible lactation support, this outcome may show more consistent and clinically meaningful improvement.

The very high heterogeneity (I^2^ ≈ 95–99%) observed across continuous outcomes reflects genuine clinical and methodological diversity among included trials (10, 12, 13). Intervention bundles differed in the number and intensity of components (e.g., type of analgesia—TAP block, intrathecal morphine, or multimodal oral regimens; timing of first ambulation; inclusion of thermal or lactation support). Perioperative policies such as anesthetic technique, urinary catheter removal, and oral intake timing also varied. Measurement windows (rest vs. activity VAS, “24 h” defined as 18–30 h) and outcome definitions further contributed. While this heterogeneity limits the precision of pooled effect sizes, the direction of effect consistently favored multimodal care. Future studies employing component network meta-analysis or multi-arm factorial designs could disentangle the contribution of individual domains. Moreover, standardized reporting of core outcomes—pain context/timepoint, mobility milestones, gastrointestinal recovery—would enhance comparability and external validity. Given that all included trials were conducted in China and most lacked blinding, the certainty of evidence is moderate to low. Therefore, these findings should be interpreted as suggestive rather than definitive.

Future work should prioritize component-level evaluation using multi-arm randomized designs or component network meta-analysis to identify high-yield elements and test whether “more components” translate to greater benefit or diminishing returns. Finally, lactation-focused add-ons tested within multimodal pathways could determine whether targeted support consistently improve breastfeeding outcomes. In addition, future studies are encouraged to incorporate validated and standardized psychological assessment tools to ensure homogenous data collection and enable meaningful comparisons or meta-analyses regarding psychological recovery. Overall, multimodal intervention bundles appear to deliver safer, faster early recovery after cesarean section; refining bundle design, standardizing outcomes, and broadening validation will be the keys to maximizing patient-centered benefits.

## Data Availability

The original contributions presented in the study are included in the article/Supplementary material, further inquiries can be directed to the corresponding author.
