# Supplementary material for: A meta-analysis of the effects of multimodal intervention measures on the recovery of postpartum women after cesarean section
Source: Front Med (Lausanne). 2025 Dec 17;12:1690139. doi: 10.3389/fmed.2025.1690139 (PMC12753421; doi:10.3389/fmed.2025.1690139)
Supplement: Supplementary file 1 [file Supplementary_file_1.docx]

**Detailed Search Strategies for Each Database**

1、PubMed

("Cesarean Section"[MeSH] OR "cesarean section" OR "cesarean delivery" OR "c-section") AND ("Multimodal Intervention" OR "Enhanced Recovery" OR "Enhanced Recovery After Surgery" OR "ERAS" OR "bundled care" OR "care pathway") AND ("Pain Management"[MeSH] OR "analgesia" OR "opioid-sparing" OR "preventive analgesia") AND ("early ambulation" OR "early mobilization") AND ("early oral intake" OR "nutrition" OR "feeding") AND ("breastfeeding support" OR "lactation" OR "mother-infant bonding") AND ("psychological support" OR "education") AND ("nursing care" OR "structured nursing")

2、Embase

('cesarean section'/exp OR 'cesarean section':ti,ab OR 'c-section') AND ('multimodal intervention' OR 'enhanced recovery'/exp OR 'enhanced recovery':ti,ab OR 'care bundle') AND ('pain management'/exp OR 'analgesia':ti,ab OR 'preventive analgesia') AND ('early mobilization' OR 'early ambulation') AND ('nutrition'/exp OR 'oral intake' OR 'feeding') AND ('breastfeeding support' OR 'psychological support' OR 'nursing intervention')

3、Web of Science

| TS=(cesarean section OR cesarean delivery OR c-section) AND TS=(multimodal intervention OR enhanced recovery OR ERAS OR bundled care) AND TS=(analgesia OR pain management OR opioid-sparing OR early ambulation OR mobilization OR nutrition OR breastfeeding OR psychological support OR nursing)  4、Scopus  TITLE-ABS-KEY ("cesarean section" OR "cesarean delivery" OR "c-section") AND TITLE-ABS-KEY ("multimodal intervention" OR "enhanced recovery" OR "ERAS" OR "care bundle") AND TITLE-ABS-KEY ("analgesia" OR "pain control" OR "opioid-sparing" OR "early ambulation" OR "nutrition" OR "feeding" OR "breastfeeding" OR "psychological education" OR "nursing care") |
| --- |

5、CNKI / Wanfang / VIP

(剖宫产 OR 剖宫分娩) AND (多模式干预 OR 加速康复外科 OR ERAS OR 综合干预 OR 护理路径) AND (镇痛 OR 疼痛管理 OR 预防性镇痛) AND (早期活动 OR 下床活动) AND (营养支持 OR 早期进食 OR 哺乳 OR 心理干预 OR 护理
